# Concomitant double-fusion of *PLEKHA7-ALK* and* INPP5D-ALK* reveals favorable alectinib sensitivity in lung adenocarcinoma: a case report and literature review

**DOI:** 10.1007/s12672-024-00899-0

**Published:** 2024-02-20

**Authors:** Pei Li, Xiao Ju, Guangjian Yang

**Affiliations:** 1 Department of Respiratory Medical Oncology, Shandong Cancer Hospital and Institute, Shandong First Medical University and Shandong Academy of Medical Sciences, No.440 Jiyan Road, Jinan, 250117 People’s Republic of China; 2 Department of Radiation Oncology, Shandong Cancer Hospital and Institute, Shandong First Medical University and Shandong Academy of Medical Sciences, No.440 Jiyan Road, Jinan, 250117 People’s Republic of China

**Keywords:** Double-fusion, PLEKHA7-ALK, INPP5D-ALK, Alectinib, Lung adenocarcinoma

## Abstract

Anaplastic lymphoma kinase (*ALK*) gene fusion is a classic driver mutation in non-small cell lung cancer (NSCLC); however, *ALK* double-fusion variants in NSCLC have rarely been reported. In this study, we reported a case with extremely uncommon *ALK* double-fusion variants. A 32-year-old female diagnosed with lung adenocarcinoma, who had developed multiple intrapulmonary and brain metastases, experienced worsening of her condition despite undergoing prior chemotherapy. Subsequent testing using next-generation sequencing (NGS) detected the presence of *PLEKHA7-ALK* and *INPP5D-ALK* double-fusion*.* The prescription of alectinib revealed potent efficacy and resulted in an increase in the survival rate. This case presented two uncommon and concomitant *ALK* fusion partners in NSCLC; more importantly, the *INPP5D-ALK* subtype has not been reported, therefore this study broadens the spectrum of *ALK* double-fusion variants and provides insight into the use of *ALK* inhibitors for the treatment of NSCLC in patients with double *ALK* fusions.

## Introduction

*ALK* gene fusion is a well-known driver mutation present in 3–7% of cases diagnosed with non-small cell lung cancer (NSCLC) [1]. Echinoderm microtubule-associated protein-like 4 (*EML4*) is the most common *ALK* fusion partner, accounting for over 80% of fusion subtypes. The deep coverage of next-generation sequencing (NGS) for *ALK* fusion partners (over 90 types) increased between 2007 and 2020, and *ALK* tyrosine kinase inhibitors (TKIs) exhibited potent activities [2]. In addition, several *ALK* double-fusion variants have been occasionally discovered and reported in cases with NSCLC [3–5]. Of note, these rare double-fusion partners might result in discrepant responses to *ALK* inhibitors, and further evidence would be urgently needed in this field. Here, we reported a case with concomitant *ALK* rare double-fusion, namely *PLEKHA7-ALK* and *INPP5D-ALK* in a patient diagnosed with lung adenocarcinoma who showed favorable response to alectinib following chemotherapy failure.

## Case presentation

**Fig. 1:**
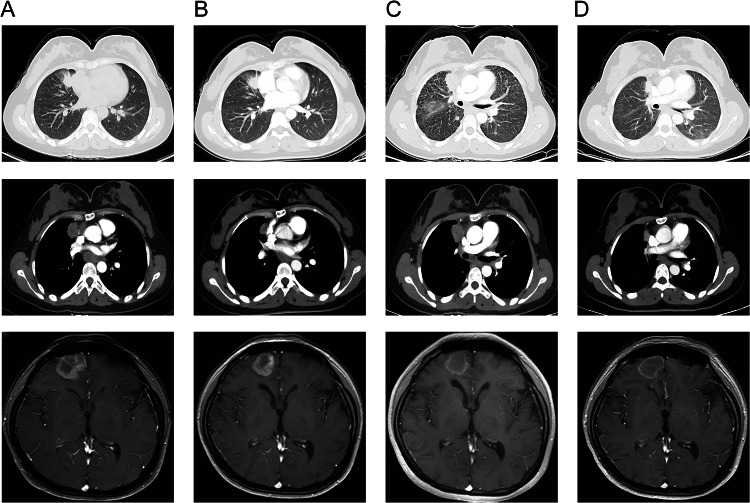
Multiple intrapulmonary and brain metastases at baseline before treatment (**A**); partial response to chemotherapy plus bevacizumab after two cycles of treatment (**B**); progression of intrapulmonary metastasis and emergence of lymphangitis carcinomatosis (**C**); partial response to alectinib with significant decrease of intrapulmonary metastasis and lymphangitis carcinomatosis (**D**)

A 32-year-old female with no smoking history was diagnosed with a mass in the upper lobe of the right lung with bilateral multiple intrapulmonary and brain metastases in July 2020 (Fig. 1A). Subsequent pulmonary puncture biopsy demonstrated lung adenocarcinoma, showing strongly positive expression of TTF-1 and Napsin A according to immunohistochemical analysis. Polymerase chain reaction (PCR) assay via tumor tissue found no alteration in *EGFR*, *ALK* and *ROS-1* genes when assayed in the resected lung tumor*.* Since July 2020, she underwent whole brain radiotherapy (WBRT) at a dose of 36 Gy/1.8 Gy/20f, along with a simultaneous integrated boost (SIB) of up to 56 Gy/20f using the intensity modulated radiation therapy (IMRT) technique. Also, she underwent chemotherapy with pemetrexed plus carboplatin, combined with an antiangiogenic agent, bevacizumab. Following two cycles of chemotherapy, she developed a partial response (PR), as shown in Fig. 1B. She experienced PR after maintenance therapy with pemetrexed and bevacizumab started after six cycles of treatment.

**Fig. 2:**
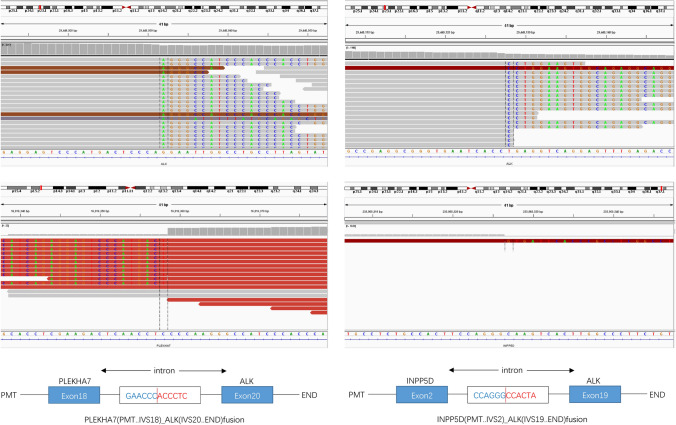
Concomitant *ALK* double-fusion *PLEKHA7-ALK* and *INPP5D-ALK* detected by NGS testing

In November 2022, her intrapulmonary metastasis significantly increased, and she developed lymphangitis carcinomatosis, while the brain metastasis remained stable (Fig. 1C). She underwent clinical examinations to monitor disease progression and achieved a progression-free survival (PFS) of 27 months without any further advancement. Owing to the outbreak of COVID-19 in China in December 2022, the patient discontinued her treatment regimen. In February 2023, two months later, the patient developed bilateral pleural metastasis, which resulted in a worsening of her dyspnea. Subsequently, she underwent pleural space drainage, and the presence of malignant adenocarcinoma cells was confirmed. The NGS analysis was carried out in Geneseeq Technology Inc. using a 6-gene targeted panel (*EGFR*, *ALK*, *ROS-1*, *BRAF*, *KRAS*, and *HER2*), which identified the simultaneous presence of two fusion genes *PLEKHA7-ALK* (P18:A20, allelic frequency: 16.60%) and *INPP5D-ALK* (I2:A19, allelic frequency: 13.92%) in the pleural effusion sample (Fig. 2). Finally, alectinib was administered since April 2023. Notably, both the multiple intrapulmonary metastases and lymphangitis carcinomatosis significantly decreased after only one month of targeted therapy (Fig. 1D). She continued alectinib until December 2023, with a favorable PR and a significant 8-month PFS benefit..

## Discussion

This is the first case presenting a rare concomitant *ALK* double-fusion, namely *PLEKHA7-ALK* and *INPP5D-ALK*, in a patient with lung adenocarcinoma, who exhibited favorable sensitivity to alectinib. Due to the low incidence, research devoted to double *ALK* fusions is extremely lacking. It is critical to determine the activity of different *ALK* fusion partners in response to different *ALK* TKIs in order to make personalized therapeutic decisions. According to the previous evidence, only several cases with distinct *ALK* double-fusions have been reported (Table 1), and crizotinib and alectinib showed exceptional activities [3–17].

**Table 1:** Summary of the characteristics of *ALK* double-fusion subtypes in previous case reports

Ref	Year	Age	Gender	*ALK* double-fusion subtypes	Efficacy of *ALK* inhibitors (response, PFS)
8	2018	56	Male	*FBXO11-ALK, EML6-ALK*	Crizotinib (PR, 11 mos)
9	2018	44	Male	*DYSF-ALK, ITGAV-ALK*	Crizotinib (SD, 3 mons)
6	2019	44	Male	*PRKCB-ALK, EML4-ALK*	Crizotinib (PR, 5 mos)
7	2019	29	Male	*BCL11A-ALK, EML4-ALK*	Crizotinib (PR, 13 mos)
3	2020	32	Male	*CCNY-ALK, ATIC-ALK*	Crizotinib (PR, 6 mos)
4	2020	64	Female	*NLRC4-ALK, EML4‐ALK*	Crizotinib (SD, 10 mos)
13	2020	55	Female	*CDK15-ALK, EML4-ALK*	Crizotinib (PR, 23 mos)
14	2020	60	Male	*BIRC6-ALK, EML4-ALK*	Alectinib (SD, 3 + mos)
15	2021	39	Female	*NBEA-ALK, EML4-ALK*	Alectinib (SD, 11mos)
16	2021	29	Female	*PDK1-ALK, STRN-ALK*	Alectinib (PR, 7mos)
5	2022	38	Female	*SSH2-ALK, EML4-ALK*	Crizotinib (PR, 4 mos)
5	2022	58	Female	*ARID2-ALK, EML4-ALK*	Crizotinib (SD, 12 mos)
10	2023	46	Male	*PPFIA1-ALK, C2orf91(intergenic)-ALK*	Alectinib (PR, 12mos)
11	2023	30	Female	*STRN-ALK, NBEA-ALK*	N/A
12	2023	28	Male	*SETD2ALK, EML4-ALK*	Alectinib (SD, 26mos)
17	2023	80	Male	*MRPL13-ALK, PPP1CB-ALK* (Acquired resistance to osimertinib)	Osimertinib with crizotinib (died after 1 month)

SD, stable disease; PR, partial disease; mos: months; N/A: not available

Here, we identified novel and concomitant *ALK* double-fusion partners, namely *PLEKHA7-ALK* and *INPP5D-ALK*, in a patient with NSCLC. The *PLEKHA7-ALK* fusion was first reported as an acquired drug resistance in a patient with lung cancer who had failed to adequately respond to osimertinib. When osimertinib and alectinib were subsequently administered in combination, it achieved a dramatic and confirmed PR with a 6-month duration of response [18]. In addition, the *PLEKHA7-ALK* fusion was also reported in renal cell carcinoma [19]. The pleckstrin homology domaincontaining A7 gene (*PLEKHA7*) encodes an adherens junction protein which is highly preserved in epithelial cells [20]. The *PLEKHA7- ALK* fusion protein remained the pleckstrin homology domain of *PLEKHA7* and the kinase domain of *ALK*. Notably, the pleckstrin homology domain might further facilitate the dimerization and activation of the *ALK* kinase [21]. To the best of our knowledge, the *INPP5D-ALK* fusion has not been reported in lung cancer to date. The inositol polyphosphate-5-phosphatase D gene (*INPP5D*) is a risk-conferring gene for Alzheimer’s disease (AD) specifically expressed in microglia in the brain, and negatively regulates the phosphoinositide 3-kinase pathway controlling cell migration, proliferation, and survival [22, 23]. The present case demonstrated the rearrangement in exon 18 of the *PLEKHA7* gene and exon 20 of the *ALK* gene, as well as the fusion of *INPP5D* (exon 2) and *ALK* (exon 19), forming a double-fusion variant *PLEKHA7-ALK* and *INPP5D-ALK*. Although there is no direct evidence to support *INPP5D-ALK* as a driver mutation, it is worth considering that *INPP5D-ABL1* fusion has exhibited sensitivity to imatinib in a patient with acute lymphoblastic leukemia [24]. Therefore, it is possible that *INPP5D-ALK* fusion could be a driver mutation in our case of NSCLC. The patient in our case showed partial response to alectinib when her disease progressed following previous chemotherapy. Despite the presence of a rare *ALK* double-fusion, this patient demonstrated a significant partial response and has benefited from alectinib for the past eight months in terms of PFS. Our findings, in this case, lack explicit research support; therefore, we can only speculate that the rare *ALK* double-fusion *PLEKHA7-ALK* and *INPP5D-ALK* might provide a promising response to the *ALK* inhibitor alectinib.

The findings of the worldwide phase III ALEX trial indicate that alectinib and crizotinib provide comparable benefits in terms of PFS, objective response rate (ORR), and duration of response (DOR) for patients with *EML4-ALK* variants 1 and 3a/b when used as the first-line treatement [25]. Although the available evidence on the association between variant types and treatment outcomes in the ALEX clinical trial is limited, patients treated with alectinib demonstrate superior response and improved survival compared to those treated with crizotinib in treatment-naive patients with *ALK*-positive NSCLC, irrespective of the *EML4-ALK* variant. However, the *ALK* double-fusion remains rare, with only a few case reports being published owing to its low incidence and detection rate. Over the past five years, we conducted a thorough search and identified 16 distinct types of *ALK* double-fusion variants. The characteristics of these variants, as reported in these case reports, are summarized in Table 1. Among them, more than half (9/16) are *EML4-ALK* together with another rare *ALK* fusion type. Crizotinib and alectinib demonstrated a positive response and significant PFS improvement for patients carrying these heterogeneous *ALK* double-fusion variants. In addition, the *ALK* double-fusion partners, namely *MRPL13-ALK* and *PPP1CB-ALK*, were reported as acquired resistant variants to *EGFR*-TKI treatment. According to these 16 case reports examined, we could not find valuable information regarding differences in patient background, treatment efficacy, and prognosis between double-fusion and single-fusion variants. However, both crizotinib and alectinib showed potent activity and a favorable survival rate for these rare *ALK* double-fusion variants.

In conclusion, this case report described a novel *ALK* double-fusion involving *PLEKHA7-ALK* and *INPP5D-ALK* in lung adenocarcinoma. Also, alectinib demonstrated potent activity and improved survival outcomes for this rather rare variant. In addition, the NGS testing provides a reliable insight for the identification of novel fusion partners for *ALK*-rearranged NSCLC.

## Data Availability

All data generated or analysed are included in this published article.
